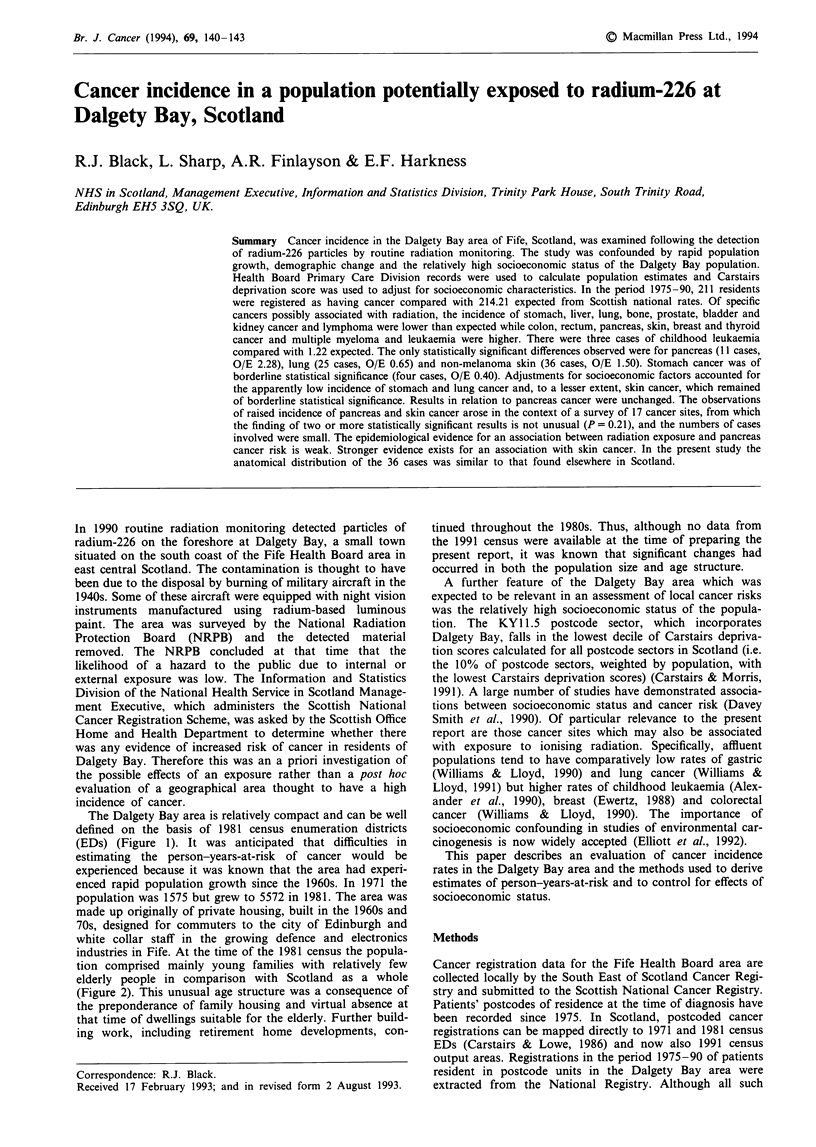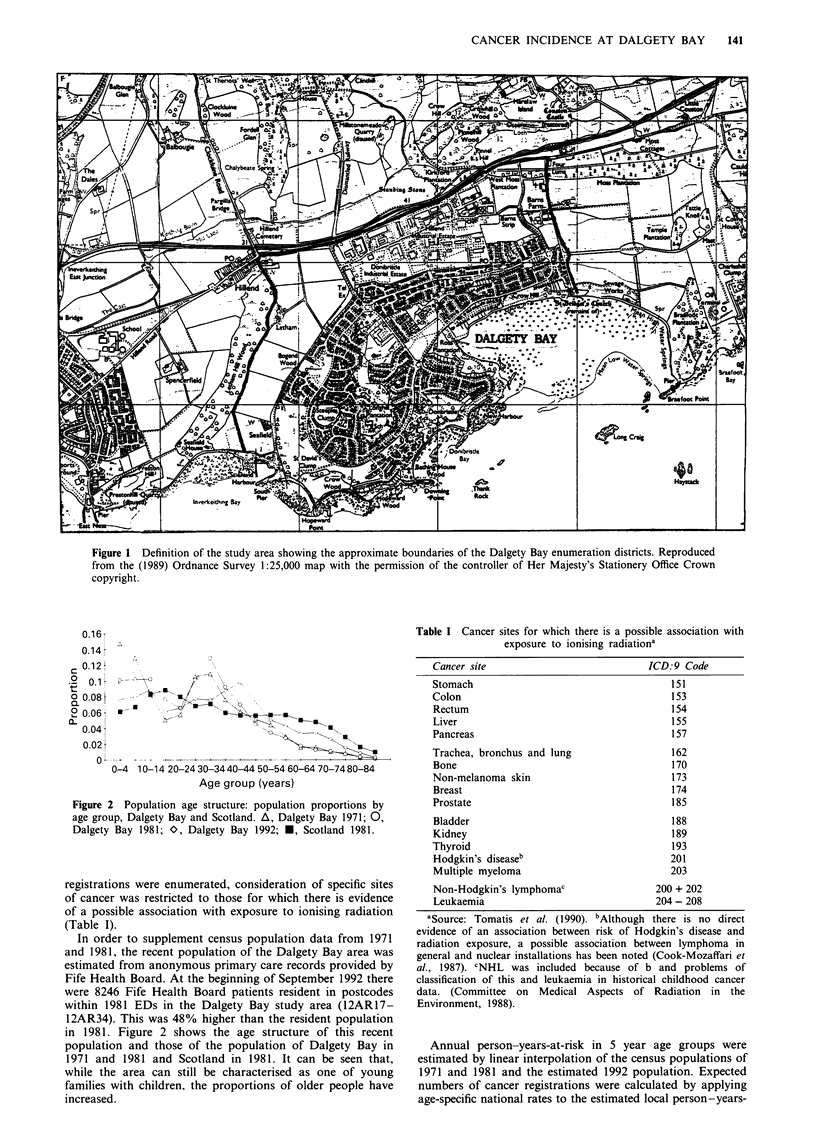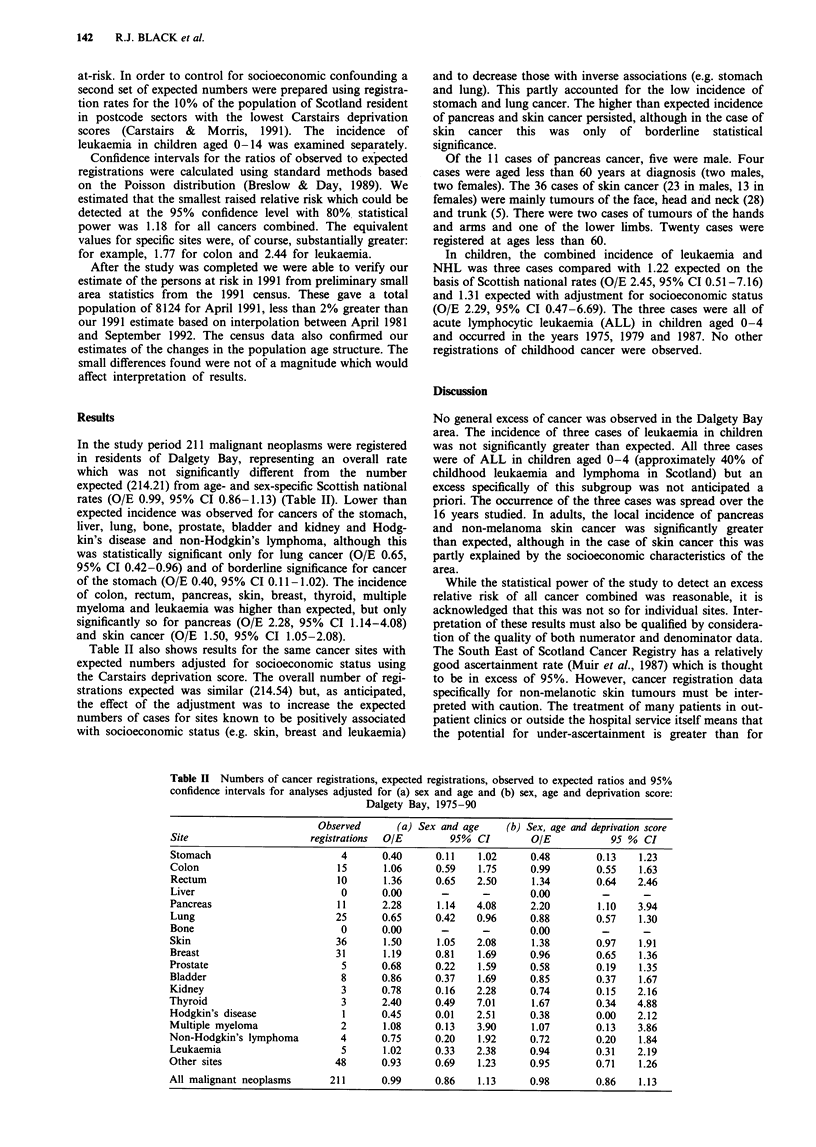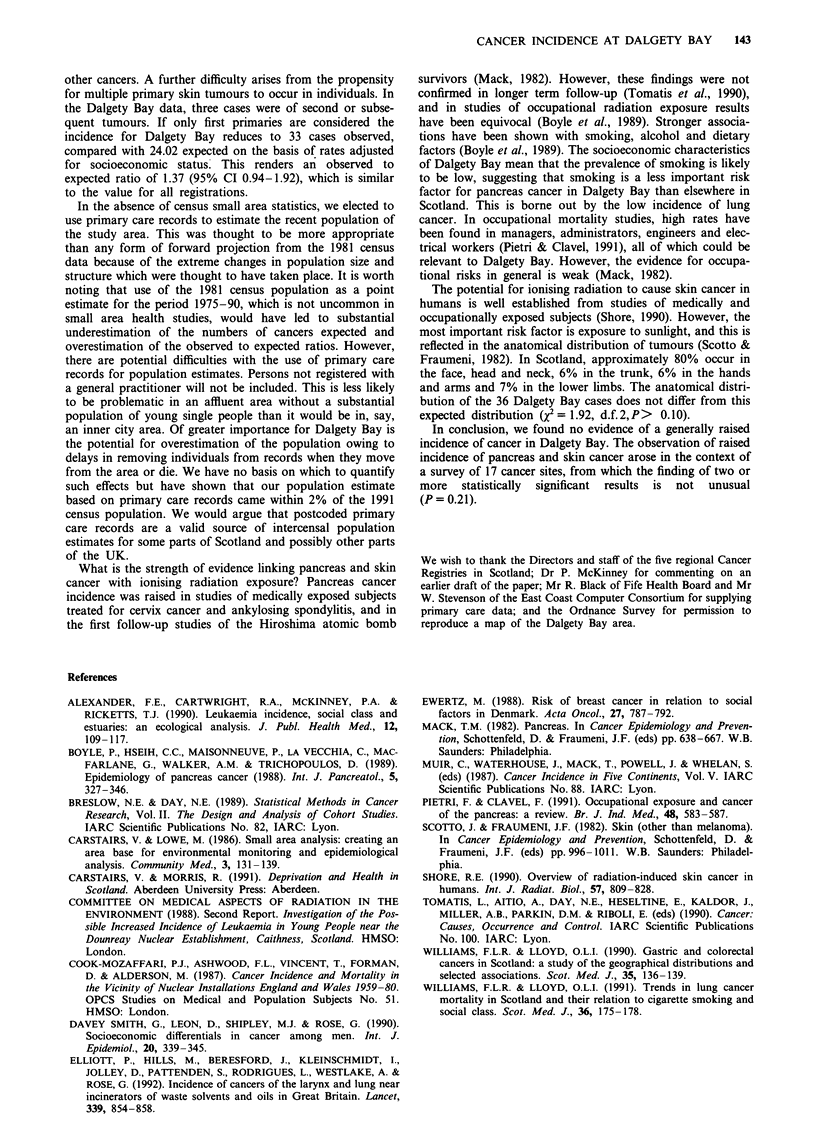# Cancer incidence in a population potentially exposed to radium-226 at Dalgety Bay, Scotland.

**DOI:** 10.1038/bjc.1994.23

**Published:** 1994-01

**Authors:** R. J. Black, L. Sharp, A. R. Finlayson, E. F. Harkness

**Affiliations:** NHS in Scotland, Information and Statistics Division, Edinburgh, UK.

## Abstract

Cancer incidence in the Dalgety Bay area of Fife, Scotland, was examined following the detection of radium-226 particles by routine radiation monitoring. The study was confounded by rapid population growth, demographic change and the relatively high socioeconomic status of the Dalgety Bay population. Health Board Primary Care Division records were used to calculate population estimates and Carstairs deprivation score was used to adjust for socioeconomic characteristics. In the period 1975-90, 211 residents were registered as having cancer compared with 214.21 expected from Scottish national rates. Of specific cancers possibly associated with radiation, the incidence of stomach, liver, lung, bone, prostate, bladder and kidney cancer and lymphoma were lower than expected while colon, rectum, pancreas, skin, breast and thyroid cancer and multiple myeloma and leukaemia were higher. There were three cases of childhood leukaemia compared with 1.22 expected. The only statistically significant differences observed were for pancreas (11 cases, O/E 2.28), lung (25 cases, O/E 0.65) and non-melanoma skin (36 cases, O/E 1.50). Stomach cancer was of borderline statistical significance (four cases, O/E 0.40). Adjustments for socioeconomic factors accounted for the apparently low incidence of stomach and lung cancer and, to a lesser extent, skin cancer, which remained of borderline statistical significance. Results in relation to pancreas cancer were unchanged. The observations of raised incidence of pancreas and skin cancer arose in the context of a survey of 17 cancer sites, from which the finding of two or more statistically significant results is not unusual (P = 0.21), and the numbers of cases involved were small. The epidemiological evidence for an association between radiation exposure and pancreas cancer risk is weak. Stronger evidence exists for an association with skin cancer. In the present study the anatomical distribution of the 36 cases was similar to that found elsewhere in Scotland.


Br. J. Cancer (1994), 69, 140-143 © Macmillan Press Ltd., 1994

### Cancer incidence in a population potentially exposed to radium-226 at Dalgety Bay, Scotland

R.J. Black, L. Sharp, A.R. Finlayson & E.F. Harkness

*NHS in Scotland, Management Executive, Information and Statistics Division, Trinity Park House, South Trinity Road, Edinburgh EH5 3SQ, UK.*

**Summary** Cancer incidence in the Dalgety Bay area of Fife, Scotland, was examined following the detection of radium-226 particles by routine radiation monitoring. The study was confounded by rapid population growth, demographic change and the relatively high socioeconomic status of the Dalgety Bay population. Health Board Primary Care Division records were used to calculate population estimates and Carstairs deprivation score was used to adjust for socioeconomic characteristics. In the period 1975-90, 211 residents were registered as having cancer compared with 214.21 expected from Scottish national rates. Of specific cancers possibly associated with radiation, the incidence of stomach, liver, lung, bone, prostate, bladder and kidney cancer and lymphoma were lower than expected while colon, rectum, pancreas, skin, breast and thyroid cancer and multiple myeloma and leukaemia were higher. There were three cases of childhood leukaemia compared with 1.22 expected. The only statistically significant differences observed were for pancreas (11 cases, O/E 2.28), lung (25 cases, O/E 0.65) and non-melanoma skin (36 cases, O/E 1.50). Stomach cancer was of borderline statistical significance (four cases, O/E 0.40). Adjustments for socioeconomic factors accounted for the apparently low incidence of stomach and lung cancer and, to a lesser extent, skin cancer, which remained of borderline statistical significance. Results in relation to pancreas cancer were unchanged. The observations of raised incidence of pancreas and skin cancer arose in the context of a survey of 17 cancer sites, from which the finding of two or more statistically significant results is not unusual ($P = 0.21$), and the numbers of cases involved were small. The epidemiological evidence for an association between radiation exposure and pancreas cancer risk is weak. Stronger evidence exists for an association with skin cancer. In the present study the anatomical distribution of the 36 cases was similar to that found elsewhere in Scotland.

In 1990 routine radiation monitoring detected particles of radium-226 on the foreshore at Dalgety Bay, a small town situated on the south coast of the Fife Health Board area in east central Scotland. The contamination is thought to have been due to the disposal by burning of military aircraft in the 1940s. Some of these aircraft were equipped with night vision instruments manufactured using radium-based luminous paint. The area was surveyed by the National Radiation Protection Board (NRPB) and the detected material removed. The NRPB concluded at that time that the likelihood of a hazard to the public due to internal or external exposure was low. The Information and Statistics Division of the National Health Service in Scotland Management Executive, which administers the Scottish National Cancer Registration Scheme, was asked by the Scottish Office Home and Health Department to determine whether there was any evidence of increased risk of cancer in residents of Dalgety Bay. Therefore this was an a priori investigation of the possible effects of an exposure rather than a *post hoc* evaluation of a geographical area thought to have a high incidence of cancer.

The Dalgety Bay area is relatively compact and can be well defined on the basis of 1981 census enumeration districts (EDs) (Figure 1). It was anticipated that difficulties in estimating the person-years-at-risk of cancer would be experienced because it was known that the area had experienced rapid population growth since the 1960s. In 1971 the population was 1575 but grew to 5572 in 1981. The area was made up originally of private housing, built in the 1960s and 70s, designed for commuters to the city of Edinburgh and white collar staff in the growing defence and electronics industries in Fife. At the time of the 1981 census the population comprised mainly young families with relatively few elderly people in comparison with Scotland as a whole (Figure 2). This unusual age structure was a consequence of the preponderance of family housing and virtual absence at that time of dwellings suitable for the elderly. Further building work, including retirement home developments, continued throughout the 1980s. Thus, although no data from the 1991 census were available at the time of preparing the present report, it was known that significant changes had occurred in both the population size and age structure.

A further feature of the Dalgety Bay area which was expected to be relevant in an assessment of local cancer risks was the relatively high socioeconomic status of the population. The KY11.5 postcode sector, which incorporates Dalgety Bay, falls in the lowest decile of Carstairs deprivation scores calculated for all postcode sectors in Scotland (i.e. the 10% of postcode sectors, weighted by population, with the lowest Carstairs deprivation scores) (Carstairs & Morris, 1991). A large number of studies have demonstrated associations between socioeconomic status and cancer risk (Davey Smith *et al.*, 1990). Of particular relevance to the present report are those cancer sites which may also be associated with exposure to ionising radiation. Specifically, affluent populations tend to have comparatively low rates of gastric (Williams & Lloyd, 1990) and lung cancer (Williams & Lloyd, 1991) but higher rates of childhood leukaemia (Alexander *et al.*, 1990), breast (Ewertz, 1988) and colorectal cancer (Williams & Lloyd, 1990). The importance of socioeconomic confounding in studies of environmental carcinogenesis is now widely accepted (Elliott *et al.*, 1992).

This paper describes an evaluation of cancer incidence rates in the Dalgety Bay area and the methods used to derive estimates of person-years-at-risk and to control for effects of socioeconomic status.

### Methods

Cancer registration data for the Fife Health Board area are collected locally by the South East of Scotland Cancer Registry and submitted to the Scottish National Cancer Registry. Patients' postcodes of residence at the time of diagnosis have been recorded since 1975. In Scotland, postcoded cancer registrations can be mapped directly to 1971 and 1981 census EDs (Carstairs & Lowe, 1986) and now also 1991 census output areas. Registrations in the period 1975-90 of patients resident in postcode units in the Dalgety Bay area were extracted from the National Registry. Although all such

Correspondence: R.J. Black.
Received 17 February 1993; and in revised form 2 August 1993.

**Figure 1** Definition of the study area showing the approximate boundaries of the Dalgety Bay enumeration districts. Reproduced from the (1989) Ordnance Survey 1:25,000 map with the permission of the controller of Her Majesty's Stationery Office Crown copyright.

**Figure 2** Population age structure: population proportions by age group, Dalgety Bay and Scotland. △, Dalgety Bay 1971; ○, Dalgety Bay 1981; ◇, Dalgety Bay 1992; ■, Scotland 1981.

registrations were enumerated, consideration of specific sites of cancer was restricted to those for which there is evidence of a possible association with exposure to ionising radiation (Table I).

In order to supplement census population data from 1971 and 1981, the recent population of the Dalgety Bay area was estimated from anonymous primary care records provided by Fife Health Board. At the beginning of September 1992 there were 8246 Fife Health Board patients resident in postcodes within 1981 EDs in the Dalgety Bay study area (12AR17–12AR34). This was 48% higher than the resident population in 1981. Figure 2 shows the age structure of this recent population and those of the population of Dalgety Bay in 1971 and 1981 and Scotland in 1981. It can be seen that, while the area can still be characterised as one of young families with children, the proportions of older people have increased.

**Table I** Cancer sites for which there is a possible association with exposure to ionising radiation[a]

| *Cancer site* | *ICD:9 Code* |
|---|---|
| Stomach | 151 |
| Colon | 153 |
| Rectum | 154 |
| Liver | 155 |
| Pancreas | 157 |
| Trachea, bronchus and lung | 162 |
| Bone | 170 |
| Non-melanoma skin | 173 |
| Breast | 174 |
| Prostate | 185 |
| Bladder | 188 |
| Kidney | 189 |
| Thyroid | 193 |
| Hodgkin's disease[b] | 201 |
| Multiple myeloma | 203 |
| Non-Hodgkin's lymphoma[c] | 200 + 202 |
| Leukaemia | 204 – 208 |

[a]Source: Tomatis *et al.* (1990). [b]Although there is no direct evidence of an association between risk of Hodgkin's disease and radiation exposure, a possible association between lymphoma in general and nuclear installations has been noted (Cook-Mozaffari *et al.*, 1987). [c]NHL was included because of b and problems of classification of this and leukaemia in historical childhood cancer data. (Committee on Medical Aspects of Radiation in the Environment, 1988).

Annual person–years-at-risk in 5 year age groups were estimated by linear interpolation of the census populations of 1971 and 1981 and the estimated 1992 population. Expected numbers of cancer registrations were calculated by applying age-specific national rates to the estimated local person–yearsat-risk. In order to control for socioeconomic confounding a second set of expected numbers were prepared using registration rates for the 10% of the population of Scotland resident in postcode sectors with the lowest Carstairs deprivation scores (Carstairs & Morris, 1991). The incidence of leukaemia in children aged 0–14 was examined separately.

Confidence intervals for the ratios of observed to expected registrations were calculated using standard methods based on the Poisson distribution (Breslow & Day, 1989). We estimated that the smallest raised relative risk which could be detected at the 95% confidence level with 80% statistical power was 1.18 for all cancers combined. The equivalent values for specific sites were, of course, substantially greater: for example, 1.77 for colon and 2.44 for leukaemia.

After the study was completed we were able to verify our estimate of the persons at risk in 1991 from preliminary small area statistics from the 1991 census. These gave a total population of 8124 for April 1991, less than 2% greater than our 1991 estimate based on interpolation between April 1981 and September 1992. The census data also confirmed our estimates of the changes in the population age structure. The small differences found were not of a magnitude which would affect interpretation of results.

### Results

In the study period 211 malignant neoplasms were registered in residents of Dalgety Bay, representing an overall rate which was not significantly different from the number expected (214.21) from age- and sex-specific Scottish national rates (O/E 0.99, 95% CI 0.86–1.13) (Table II). Lower than expected incidence was observed for cancers of the stomach, liver, lung, bone, prostate, bladder and kidney and Hodgkin's disease and non-Hodgkin's lymphoma, although this was statistically significant only for lung cancer (O/E 0.65, 95% CI 0.42–0.96) and of borderline significance for cancer of the stomach (O/E 0.40, 95% CI 0.11–1.02). The incidence of colon, rectum, pancreas, skin, breast, thyroid, multiple myeloma and leukaemia was higher than expected, but only significantly so for pancreas (O/E 2.28, 95% CI 1.14–4.08) and skin cancer (O/E 1.50, 95% CI 1.05–2.08).

Table II also shows results for the same cancer sites with expected numbers adjusted for socioeconomic status using the Carstairs deprivation score. The overall number of registrations expected was similar (214.54) but, as anticipated, the effect of the adjustment was to increase the expected numbers of cases for sites known to be positively associated with socioeconomic status (e.g. skin, breast and leukaemia) and to decrease those with inverse associations (e.g. stomach and lung). This partly accounted for the low incidence of stomach and lung cancer. The higher than expected incidence of pancreas and skin cancer persisted, although in the case of skin cancer this was only of borderline statistical significance.

Of the 11 cases of pancreas cancer, five were male. Four cases were aged less than 60 years at diagnosis (two males, two females). The 36 cases of skin cancer (23 in males, 13 in females) were mainly tumours of the face, head and neck (28) and trunk (5). There were two cases of tumours of the hands and arms and one of the lower limbs. Twenty cases were registered at ages less than 60.

In children, the combined incidence of leukaemia and NHL was three cases compared with 1.22 expected on the basis of Scottish national rates (O/E 2.45, 95% CI 0.51–7.16) and 1.31 expected with adjustment for socioeconomic status (O/E 2.29, 95% CI 0.47–6.69). The three cases were all of acute lymphocytic leukaemia (ALL) in children aged 0–4 and occurred in the years 1975, 1979 and 1987. No other registrations of childhood cancer were observed.

### Discussion

No general excess of cancer was observed in the Dalgety Bay area. The incidence of three cases of leukaemia in children was not significantly greater than expected. All three cases were of ALL in children aged 0–4 (approximately 40% of childhood leukaemia and lymphoma in Scotland) but an excess specifically of this subgroup was not anticipated a priori. The occurrence of the three cases was spread over the 16 years studied. In adults, the local incidence of pancreas and non-melanoma skin cancer was significantly greater than expected, although in the case of skin cancer this was partly explained by the socioeconomic characteristics of the area.

While the statistical power of the study to detect an excess relative risk of all cancer combined was reasonable, it is acknowledged that this was not so for individual sites. Interpretation of these results must also be qualified by consideration of the quality of both numerator and denominator data. The South East of Scotland Cancer Registry has a relatively good ascertainment rate (Muir et al., 1987) which is thought to be in excess of 95%. However, cancer registration data specifically for non-melanotic skin tumours must be interpreted with caution. The treatment of many patients in outpatient clinics or outside the hospital service itself means that the potential for under-ascertainment is greater than for

**Table II** Numbers of cancer registrations, expected registrations, observed to expected ratios and 95% confidence intervals for analyses adjusted for (a) sex and age and (b) sex, age and deprivation score: Dalgety Bay, 1975–90

| Site | Observed registrations | (a) Sex and age O/E | 95% CI | | (b) Sex, age and deprivation score O/E | 95 % CI | |
|---|---|---|---|---|---|---|---|
| Stomach | 4 | 0.40 | 0.11 | 1.02 | 0.48 | 0.13 | 1.23 |
| Colon | 15 | 1.06 | 0.59 | 1.75 | 0.99 | 0.55 | 1.63 |
| Rectum | 10 | 1.36 | 0.65 | 2.50 | 1.34 | 0.64 | 2.46 |
| Liver | 0 | 0.00 | – | – | 0.00 | – | – |
| Pancreas | 11 | 2.28 | 1.14 | 4.08 | 2.20 | 1.10 | 3.94 |
| Lung | 25 | 0.65 | 0.42 | 0.96 | 0.88 | 0.57 | 1.30 |
| Bone | 0 | 0.00 | – | – | 0.00 | – | – |
| Skin | 36 | 1.50 | 1.05 | 2.08 | 1.38 | 0.97 | 1.91 |
| Breast | 31 | 1.19 | 0.81 | 1.69 | 0.96 | 0.65 | 1.36 |
| Prostate | 5 | 0.68 | 0.22 | 1.59 | 0.58 | 0.19 | 1.35 |
| Bladder | 8 | 0.86 | 0.37 | 1.69 | 0.85 | 0.37 | 1.67 |
| Kidney | 3 | 0.78 | 0.16 | 2.28 | 0.74 | 0.15 | 2.16 |
| Thyroid | 3 | 2.40 | 0.49 | 7.01 | 1.67 | 0.34 | 4.88 |
| Hodgkin's disease | 1 | 0.45 | 0.01 | 2.51 | 0.38 | 0.00 | 2.12 |
| Multiple myeloma | 2 | 1.08 | 0.13 | 3.90 | 1.07 | 0.13 | 3.86 |
| Non-Hodgkin's lymphoma | 4 | 0.75 | 0.20 | 1.92 | 0.72 | 0.20 | 1.84 |
| Leukaemia | 5 | 1.02 | 0.33 | 2.38 | 0.94 | 0.31 | 2.19 |
| Other sites | 48 | 0.93 | 0.69 | 1.23 | 0.95 | 0.71 | 1.26 |
| All malignant neoplasms | 211 | 0.99 | 0.86 | 1.13 | 0.98 | 0.86 | 1.13 |

other cancers. A further difficulty arises from the propensity for multiple primary skin tumours to occur in individuals. In the Dalgety Bay data, three cases were of second or subsequent tumours. If only first primaries are considered the incidence for Dalgety Bay reduces to 33 cases observed, compared with 24.02 expected on the basis of rates adjusted for socioeconomic status. This renders an observed to expected ratio of 1.37 (95% CI 0.94–1.92), which is similar to the value for all registrations.

In the absence of census small area statistics, we elected to use primary care records to estimate the recent population of the study area. This was thought to be more appropriate than any form of forward projection from the 1981 census data because of the extreme changes in population size and structure which were thought to have taken place. It is worth noting that use of the 1981 census population as a point estimate for the period 1975–90, which is not uncommon in small area health studies, would have led to substantial underestimation of the numbers of cancers expected and overestimation of the observed to expected ratios. However, there are potential difficulties with the use of primary care records for population estimates. Persons not registered with a general practitioner will not be included. This is less likely to be problematic in an affluent area without a substantial population of young single people than it would be in, say, an inner city area. Of greater importance for Dalgety Bay is the potential for overestimation of the population owing to delays in removing individuals from records when they move from the area or die. We have no basis on which to quantify such effects but have shown that our population estimate based on primary care records came within 2% of the 1991 census population. We would argue that postcoded primary care records are a valid source of intercensal population estimates for some parts of Scotland and possibly other parts of the UK.

What is the strength of evidence linking pancreas and skin cancer with ionising radiation exposure? Pancreas cancer incidence was raised in studies of medically exposed subjects treated for cervix cancer and ankylosing spondylitis, and in the first follow-up studies of the Hiroshima atomic bomb survivors (Mack, 1982). However, these findings were not confirmed in longer term follow-up (Tomatis *et al.*, 1990), and in studies of occupational radiation exposure results have been equivocal (Boyle *et al.*, 1989). Stronger associations have been shown with smoking, alcohol and dietary factors (Boyle *et al.*, 1989). The socioeconomic characteristics of Dalgety Bay mean that the prevalence of smoking is likely to be low, suggesting that smoking is a less important risk factor for pancreas cancer in Dalgety Bay than elsewhere in Scotland. This is borne out by the low incidence of lung cancer. In occupational mortality studies, high rates have been found in managers, administrators, engineers and electrical workers (Pietri & Clavel, 1991), all of which could be relevant to Dalgety Bay. However, the evidence for occupational risks in general is weak (Mack, 1982).

The potential for ionising radiation to cause skin cancer in humans is well established from studies of medically and occupationally exposed subjects (Shore, 1990). However, the most important risk factor is exposure to sunlight, and this is reflected in the anatomical distribution of tumours (Scotto & Fraumeni, 1982). In Scotland, approximately 80% occur in the face, head and neck, 6% in the trunk, 6% in the hands and arms and 7% in the lower limbs. The anatomical distribution of the 36 Dalgety Bay cases does not differ from this expected distribution ($\chi^2 = 1.92$, d.f. 2, $P > 0.10$).

In conclusion, we found no evidence of a generally raised incidence of cancer in Dalgety Bay. The observation of raised incidence of pancreas and skin cancer arose in the context of a survey of 17 cancer sites, from which the finding of two or more statistically significant results is not unusual ($P = 0.21$).

We wish to thank the Directors and staff of the five regional Cancer Registries in Scotland; Dr P. McKinney for commenting on an earlier draft of the paper; Mr R. Black of Fife Health Board and Mr W. Stevenson of the East Coast Computer Consortium for supplying primary care data; and the Ordnance Survey for permission to reproduce a map of the Dalgety Bay area.

### References

ALEXANDER, F.E., CARTWRIGHT, R.A., MCKINNEY, P.A. & RICKETTS, T.J. (1990). Leukaemia incidence, social class and estuaries: an ecological analysis. *J. Publ. Health Med.*, **12**, 109–117.

BOYLE, P., HSEIH, C.C., MAISONNEUVE, P., LA VECCHIA, C., MACFARLANE, G., WALKER, A.M. & TRICHOPOULOS, D. (1989). Epidemiology of pancreas cancer (1988). *Int. J. Pancreatol.*, **5**, 327–346.

BRESLOW, N.E. & DAY, N.E. (1989). *Statistical Methods in Cancer Research*, Vol. II. *The Design and Analysis of Cohort Studies.* IARC Scientific Publications No. 82, IARC: Lyon.

CARSTAIRS, V. & LOWE, M. (1986). Small area analysis: creating an area base for environmental monitoring and epidemiological analysis. *Community Med.*, **3**, 131–139.

CARSTAIRS, V. & MORRIS, R. (1991). *Deprivation and Health in Scotland.* Aberdeen University Press: Aberdeen.

COMMITTEE ON MEDICAL ASPECTS OF RADIATION IN THE ENVIRONMENT (1988). Second Report. *Investigation of the Possible Increased Incidence of Leukaemia in Young People near the Dounreay Nuclear Establishment, Caithness, Scotland.* HMSO: London.

COOK-MOZAFFARI, P.J., ASHWOOD, F.L., VINCENT, T., FORMAN, D. & ALDERSON, M. (1987). *Cancer Incidence and Mortality in the Vicinity of Nuclear Installations England and Wales 1959–80.* OPCS Studies on Medical and Population Subjects No. 51. HMSO: London.

DAVEY SMITH, G., LEON, D., SHIPLEY, M.J. & ROSE, G. (1990). Socioeconomic differentials in cancer among men. *Int. J. Epidemiol.*, **20**, 339–345.

ELLIOTT, P., HILLS, M., BERESFORD, J., KLEINSCHMIDT, I., JOLLEY, D., PATTENDEN, S., RODRIGUES, L., WESTLAKE, A. & ROSE, G. (1992). Incidence of cancers of the larynx and lung near incinerators of waste solvents and oils in Great Britain. *Lancet*, **339**, 854–858.

EWERTZ, M. (1988). Risk of breast cancer in relation to social factors in Denmark. *Acta Oncol.*, **27**, 787–792.

MACK, T.M. (1982). Pancreas. In *Cancer Epidemiology and Prevention*, Schottenfeld, D. & Fraumeni, J.F. (eds) pp. 638–667. W.B. Saunders: Philadelphia.

MUIR, C., WATERHOUSE, J., MACK, T., POWELL, J. & WHELAN, S. (eds) (1987). *Cancer Incidence in Five Continents*, Vol. V. IARC Scientific Publications No. 88. IARC: Lyon.

PIETRI, F. & CLAVEL, F. (1991). Occupational exposure and cancer of the pancreas: a review. *Br. J. Ind. Med.*, **48**, 583–587.

SCOTTO, J. & FRAUMENI, J.F. (1982). Skin (other than melanoma). In *Cancer Epidemiology and Prevention*, Schottenfeld, D. & Fraumeni, J.F. (eds) pp. 996–1011. W.B. Saunders: Philadelphia.

SHORE, R.E. (1990). Overview of radiation-induced skin cancer in humans. *Int. J. Radiat. Biol.*, **57**, 809–828.

TOMATIS, L., AITIO, A., DAY, N.E., HESELTINE, E., KALDOR, J., MILLER, A.B., PARKIN, D.M. & RIBOLI, E. (eds) (1990). *Cancer: Causes, Occurrence and Control.* IARC Scientific Publications No. 100. IARC: Lyon.

WILLIAMS, F.L.R. & LLOYD, O.L.I. (1990). Gastric and colorectal cancers in Scotland: a study of the geographical distributions and selected associations. *Scot. Med. J.*, **35**, 136–139.

WILLIAMS, F.L.R. & LLOYD, O.L.I. (1991). Trends in lung cancer mortality in Scotland and their relation to cigarette smoking and social class. *Scot. Med. J.*, **36**, 175–178.